# Autocatalytic Activation of the Furin Zymogen Requires Removal of the Emerging Enzyme's N-Terminus from the Active Site

**DOI:** 10.1371/journal.pone.0005031

**Published:** 2009-04-07

**Authors:** Katarzyna Gawlik, Sergey A. Shiryaev, Wenhong Zhu, Khatereh Motamedchaboki, Roxane Desjardins, Robert Day, Albert G. Remacle, Boguslaw Stec, Alex Y. Strongin

**Affiliations:** 1 Burnham Institute for Medical Research, La Jolla, California, United States of America; 2 University of Sherbrooke, Sherbrooke, Quebec, Canada; Monash University, Australia

## Abstract

**Background:**

Before furin can act on protein substrates, it must go through an ordered process of activation. Similar to many other proteinases, furin is synthesized as a zymogen (profurin) which becomes active only after the autocatalytic removal of its auto-inhibitory prodomain. We hypothesized that to activate profurin its prodomain had to be removed and, in addition, the emerging enzyme's N-terminus had to be ejected from the catalytic cleft.

**Methodology/Principal Findings:**

We constructed and analyzed the profurin mutants in which the egress of the emerging enzyme's N-terminus from the catalytic cleft was restricted. Mutants were autocatalytically processed at only the primary cleavage site Arg-Thr-Lys-Arg^107^↓Asp^108^, but not at both the primary and the secondary (Arg-Gly-Val-Thr-Lys-Arg^75^↓Ser^76^) cleavage sites, yielding, as a result, the full-length prodomain and mature furins commencing from the N-terminal Asp108. These correctly processed furin mutants, however, remained self-inhibited by the constrained N-terminal sequence which continuously occupied the S′ sub-sites of the catalytic cleft and interfered with the functional activity. Further, using the *in vitro* cleavage of the purified prodomain and the analyses of colon carcinoma LoVo cells with the reconstituted expression of the wild-type and mutant furins, we demonstrated that a three-step autocatalytic processing including the cleavage of the prodomain at the previously unidentified Arg-Leu-Gln-Arg^89^↓Glu^90^ site, is required for the efficient activation of furin.

**Conclusions/Significance:**

Collectively, our results show the restrictive role of the enzyme's N-terminal region in the autocatalytic activation mechanisms. In a conceptual form, our data apply not only to profurin alone but also to a range of self-activated proteinases.

## Introduction

A variety of proteins, including serine proteinases and metalloproteinases, growth factors, and adhesion molecules as well as bacterial and viral pathogens, is initially synthesized as inactive precursors. Specific processing is required to transform these proproteins into biologically active proteins [1]. Furin and related proprotein convertases (PCs) are specialized serine endoproteinases, which cleave the multibasic motifs R-X-R/K/X-R to transform proproteins into biologically active proteins and peptides [2]. Seven furin-family proteases (furin, PACE4, PC1/3, PC2, PC4, PC5/6 and PC7) have been identified in humans [3]. Ubiquitously expressed furin is the most studied enzyme among all of the PCs [4], [5], [6]. Structurally and functionally, furin resembles its evolutionary precursor, yeast kexin. Furin is synthesized as a pre-proprotein which contains a signal peptide, a prodomain, a subtilisin-like catalytic domain, a middle P domain, a cysteine-rich region, a transmembrane anchor and a cytoplasmic tail. The N-terminal 81-residue prodomain (residues Gln^27^-Arg^107^) functions as a potent auto-inhibitor [7], [8]. Furin and other PCs require proteolytic removal of the inhibitory prodomain to become proteolytically potent enzymes [2], [9].

No natural protein inhibitors of furin are known. The highly potent synthetic peptidic inhibitor decanoyl-Arg-Val-Lys-Arg-chloromethylketone (dec-RVKR-cmk) and α1-antitrypsin variant Portland are used to inhibit furin in cleavage reactions *in vitro* and in cell-based tests. The original α1-antitrypsin serpin is the natural inhibitor of neutrophil elastase [10]. After a natural mutation of the active site Met358 to Arg the mutant, called α1-antitrypsin variant Pittsburgh, becomes a potent inhibitor of thrombin [11]. The additional, genetically engineered, mutation at position 355 generates a mutant known as α1-antitrypsin variant Portland (α1-PDX). As a result of this additional mutation, α1-PDX exhibits a minimal furin cleavage motif (Arg^355^-Ile-Pro-Arg^358^-) in its reactive site loop and is a 0.5 nM inhibitor of furin [12], [13]. α1-PDX inhibits furin by a slow, tight-binding mechanism and, similar to other serpins, functions as a suicide substrate inhibitor. Because of these parameters, α1-PDX forms a covalent, SDS- and heat-stable, complex with functionally active furin [13].

It has been suggested that the activation of the *de novo* synthesized furin occurs through a two-step autoproteolytic mechanism leading to the cleavage of its cognate N-terminal prodomain. Only a few landmarks of furin activation are known. The folded furin zymogen initially undergoes an autocatalytic excision at the primary Arg-Thr-Lys-Arg^107^↓Asp^108^ site [14], [15]. This cleavage alone, however, is not sufficient for enzymatic activation because furin remains inhibited by its non-covalently associated prodomain [16], [17]. After the translocation of furin into the mildly acidic trans-Golgi network/endoplasmic system, the primary cleavage is followed by an autocatalytic cleavage at the secondary Arg-Gly-Val-Thr-Lys-Arg^75^↓Ser^76^ site which releases the prodomain fragments [18], [19]. As a result, the prodomain's inhibitory function is inactivated and the mature, proteolytically potent enzyme is generated. These and other studies [20], [21] were focused on the inhibitory prodomain. The functional significance of the emerging N-terminal mature furin sequence, despite its obvious role in the autocatalytic activation mechanism, remains unexplored.

We hypothesized that to render the emerging enzyme functionally active its N-terminal region had to egress from its original position in the preformed catalytic cleft of profurin. This event is in addition to the autocatalytic removal of the inhibitory prodomain. We also suspect that exhaustive proteolysis of the liberated prodomain is also required to prevent re-inhibition of the emerging, activated furin.

To support our hypothesis, we constructed and analyzed mutant furins with amino acid substitutions and insertions into the N-terminal region of the emerging enzyme and re-examined the proteolytic processing of the furin prodomain. Collectively, our biochemical experiments and *in silico* modeling determined that an ejection mechanism is necessary to accomplish the exodus of the N-terminus from the active site of the emerging enzyme. If this mechanism is inactivated and the N-terminus is not released from the catalytic cleft, profurin will be processed but the resulting enzyme will remain self-inhibited.

## Results

### Modeling of the activation mechanism

**Figure 1:**
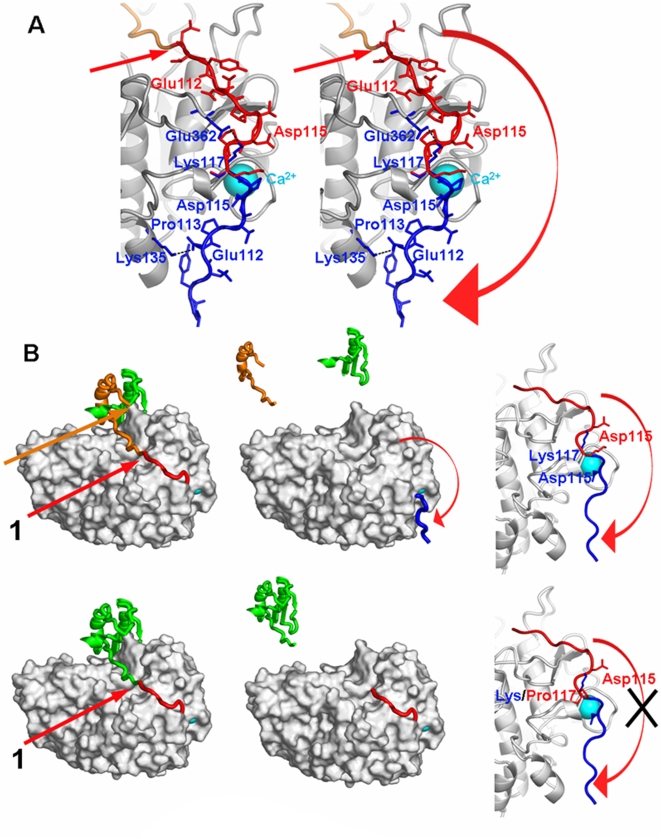
Structural model of the WT and K117P furins. (A) Stereo view of the WT furin catalytic cleft before (a red ribbon) and after (a blue ribbon) the cleavage at the primary Arg- Thr-Lys-Arg^107^↓Asp^108^ site. The red curved arrow indicates the motion of the 108–117 sequence from its position in profurin to its final position in the mature furin enzyme. The open conformation of the mature furin enzyme is stabilized by strong electrostatic interactions of Asp115 and a calcium ion (a blue sphere), and, in addition, by two salt bridges (Glu112-Lys135 and Lys117-Glu362). (B) The 26–107 prodomain and the 108–574 catalytic domain-P domain of furin are shown in green and grey, respectively. The modeled structure of the 108–117 sequence in profurin is shown in red while the actual structure of the 108–117 sequence in the mature WT furin enzyme is in blue. *Top panels* - In the structure of the WT furin, the 76–107 peptide sequence, which is generated as a result of the secondary cleavage, is shown as an orange ribbon. The arrows 1 and 2 indicate the primary and the secondary cleavage sites, respectively. *Left panel*, profurin; *middle panel*, mature furin, *right panel*, close-up. *Bottom panels* – In the K117P mutant the formation of an additional helix is necessary to eject the emerging N-terminus from the active site but it is sterically forbidden because of the adjacent Pro116. As a result, the N-end region of the mature mutant enzyme continue to occupy the S′ sub-sites leading to the inactive enzyme. The arrow 1 indicates the cleavage at the primary cleavage site alone that, as a result, generates the liberated intact prodomain (green). *Left panel*, profurin; *middle panel*, mature furin, *right panel*, close-up.

In our modeled profurin structure, the relative position of the prodomain and the catalytic domain was similar to that reported for the subtilisin-propeptide complex (Protein Data Bank code 1SCJ). The identified helix sheet interaction of the propeptide with the catalytic domain is conserved in the subtilisin family [22], [23], [24] and also in PCs including furin (Figure 1A). The 101–113 sequence region that spans the primary Arg-Thr-Lys-Arg^107^↓Asp^108^ cleavage site was positioned in the active site of profurin in a manner similar to the dec-RVKR-cmk inhibitor in the 1P8J crystal structure of murine furin [25], [26].

According to the X-ray structure of furin, the position of the N-terminal Asp108 is ∼40 Å away from the catalytic cleft [25]. In contrast, the primary cleavage site sequence Arg-Thr-Lys-Arg^107^↓Asp^108^ is located directly in the active site of the latent profurin. Through its electrostatic interactions with the calcium ion Asp115 plays a primary role in the active furin conformation by supporting the entire structural organization of the N-terminal Asp^108^-Val-Tyr-Gln-Glu-Pro-Thr-Asp-Pro-Lys^117^ region. There are also multiple hydrophobic interactions of Pro113 with the neighboring Val213, Phe118 and Trp138 residues. Two additional charge contacts (Glu112-Lys135 and Lys117-Glu362) also play an important role in the stabilization of the N-terminal 108–117 region. Both the Glu112-Lys135 and Lys117-Glu362 interactions appear to stabilize the structure of the N-terminal region of the active furin molecule (Figure 1A).

According to our modeling, the zymogen conformation leading to self-cleavage provides less favorable contacts for all charged residues. The electrostatic forces originating from broken ion bridges provide the energy that would be released as a result of the Arg-Thr-Lys-Arg^107^↓Asp^108^ site cleavage. This energy burst will stimulate repositioning of the novel N-terminus (Asp108) and will lead to the mature furin conformation where Asp108 is 40Å away from the active site. In the furin zymogen, the egress of the emerging enzyme's N-terminal region from the active site follows the autocatalytic cleavage of the primary cleavage sequence Arg-Thr-Lys-Arg^107^↓Asp^108^. To accomplish this egress, the unwinding of the 108–117 region structure is necessary. This unwinding includes a change of the backbone conformation of Lys117, the abrogation of the electrostatic contact of Asp115 with the calcium ion and also with other auxiliary electrostatic contacts and the hydrophobic interactions involving Pro113. It is highly probable that the autocatalytic cleavage at the Arg-Thr-Lys-Arg^107^↓Asp^108^ site causes Asp115 to become available for electrostatic contact with the calcium ion and, as a result, reposition the backbone to its location observed in active furin. To accommodate the ligation of Asp115 to the calcium ion, the chain reverses direction and ejects the emerging N-terminus thus liberating the multiple S′ sites of the active site of the mature, processed, furin. A similar mechanism was earlier suggested for the maturation of subtilisin BPN' [27].

According to our modeling, the N-terminal insertions in the mature furin sequence should result in an extended conformation in which the primary cleavage site Arg-Thr-Lys-Arg^107^↓Asp^108^ will access the active site without breaking the electrostatic interactions of Asp115 with the calcium ion (Figure 1B). Following the cleavage at this primary site, the N-terminal part of the emerging active furin may become partially mobile, albeit incapable of achieving the normally occurring 40 Å repositioning, and, in contrast to wild-type (WT) furin, the N-terminal region 108–117 will continue to occupy the multiple S′ sub-sites of the active site cavity. In turn, in the K117P mutant the steric properties of proline will make any significant repositioning of the N-terminal region nearly impossible, thus making the structure of the 108–117 region in the activated mature furin similar to that of profurin.

### Recombinant constructs

**Figure 2:**
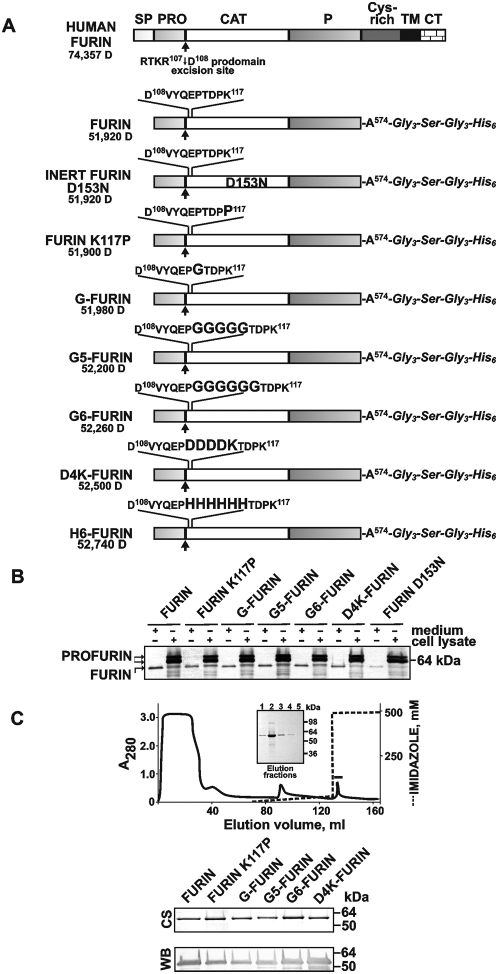
Constructs and the purification of soluble furin. (A) Furin constructs. The constructs were truncated at position Ala574 and C-terminally linked to a Gly_3_-Ser-Gly_3_ linker followed by a His_6_ tag. To generate insertion mutants, the Gly (G), Gly-Gly-Gly-Gly-Gly (G5), Gly-Gly-Gly-Gly-Gly-Gly (G6), Asp-Asp-Asp-Asp-Lys (D4K) and His-His-His-His-His-His (H6) sequences (enlarged) were inserted between positions Pro113 and Thr114 of the furin sequence. The hinge motion Lys117 and the essential Asp153 were mutated to generate the K117P and inert D153N constructs. SP, PRO, CAT, P, Cys-rich, TM and CT, signal peptide, prodomain, catalytic domain, P domain, cysteine-rich region, transmembrane domain anchor and cytoplasmic tail, respectively. The primary Arg-Thr-Lys-Arg^107^↓Asp^108^ autocatalytic cleavage site is shown by an arrow. The numbers show the molecular mass of the furin constructs. (B) Intracellular and secreted furin in Sf9 cells. Cells (2×10^6^/ml) were placed in wells of a 6-well plate and infected with the respective recombinant baculoviruses with a 1–2 multiplicity of infection. After a 36–38 h incubation at 27°C the cells and the 1 ml medium samples were collected. Cells were then lysed in 1 ml 50 mM Tris-HCl, pH 8.0, containing 1% SDS, 1 mM EDTA, 100 mM NaCl, 1 mM phenylmethyl sulfonylfluoride and protease inhibitor cocktail. Both the cell lysate and medium samples (1 µl each) were analyzed by Western blotting with the MON-148 antibody against the catalytic domain of furin. C, Purification of furin by metal-chelating chromatography. *Upper panel* - Impurities were removed using a linear gradient of 0–25 mM imidazole; furin was eluted with 500 mM imidazole. In the chromatogram, the furin-containing fractions are marked with a solid line. *Inset* - SDS-PAGE of the 500 mM imidazole fraction. *Lower panel* - SDS-PAGE (1 µg protein/lane) followed by Coomassie staining (CS) and Western blotting (WB; 100 ng protein/lane) of the purified furin samples.

To support our modeling studies experimentally and to determine the requirements for the activation of furin, WT and mutant constructs were designed. To obtain the soluble constructs, which could be readily isolated from the medium by metal-chelating chromatography, the human furin sequence was truncated at Ala574 and the truncation was then linked to a flexible 7-residue linker followed by a C-terminal His_6_ tag. To increase the distance from the N-terminus to the critical Lys117 in the N-terminal region of furin, we constructed the insertion mutants. In the insertion mutants, a single Gly residue and the Gly_5_ and Gly_6_ peptide sequences were inserted between positions Pro113 and Thr114 of the furin sequence (G, G5 and G6 mutants, respectively). To confirm that the observed effects were not unique for the Gly-containing sequences, both the positively charged His_6_ and the negatively charged Asp-Asp-Asp-Asp-Lys sequences were also inserted between Pro113 and Thr114 (H6 and D4K mutants, respectively). To determine the role of the hinge motion Lys117, the K117P mutant was also isolated. As a control, we constructed the catalytically inert D153N mutant with the substitution of the essential active site Asp153 (Figure 2A).

The constructs were then expressed in Sf9 cells infected with the recombinant baculovirus. To determine the efficiency of the profurin synthesis and the processing and secretion of mature furins, we used Western blotting of the cell lysate and conditioned medium aliquots (Figure 2B). Two profurin forms, presumably preprofurin and profurin, were predominant in the cell lysates while the processed furin alone was detected in the medium samples. With the exception of the inert D153N construct, the levels of the mutants we observed in the cell lysate and medium samples were comparable with those of the WT construct. Because the autocatalysis is mandatory for furin processing and secretion, the inert D153N was efficiently synthesized but inefficiently processed and secreted. This inefficient processing and secretion made the purification of the D153N mutant exceedingly difficult. In addition, the molecular weight of the secreted mutants was similar to that of the WT construct suggesting that the mutations did not affect the efficiency of autocatalytic processing and that the resulting secretory mutants were each capable of prodomain processing at the primary cleavage site Arg-Thr-Lys-Arg^107^↓Asp^108^.

### Isolation and the N-terminal sequence of mutant furins

We purified the mutant constructs to identify their N-terminal sequence and enzymatic features. In contrast to the inert D153N mutant, other mutants including the K117P, G, G5, G6, D4K and H6, were readily isolated from the medium using metal-chelating chromatography (Figure 2C). Because the properties of the H6- and G6-furin mutants were very similar, the first will not be discussed in the text below.

To confirm the conversion of profurin into a correctly processed, mature furin, the WT, K117P and G6 purified samples were each subjected to N-terminal microsequencing. Only one N-terminal peptide sequence (D^108^ VYQEP) that corresponded to mature furin was identified, thus confirming that the WT and the mutants were processed through an autocatalytic activation pathway involving the cleavage of profurin at the conventional primary Arg-Thr-Lys-Arg^107^↓Asp^108^ site [14], [15], [18], [19].

### Furin activity assays

**Figure 3:**
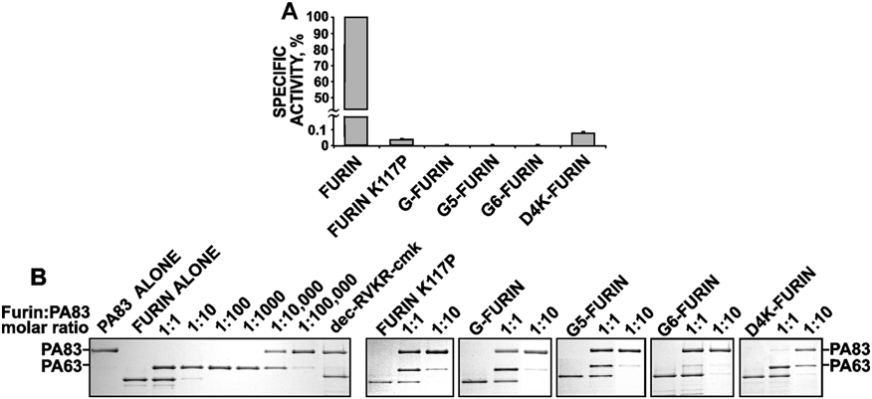
Characterization of mutant furins. (A) Specific activity of mutant furins against Abz-Arg-Val-Lys-Arg-Gly-Leu-Ala-Tyr(NO_2_)-Asp-OH. The substrate was efficiently cleaved by the WT furin (1 nM) while the mutants (1 µM) were ∼1000-less active. (B) Cleavage of anthrax PA83 by mutant furins. PA83 (1 µg) was co-incubated for 1 h at 37°C with the purified furin samples at the indicated enzyme-substrate ratio. The digest samples were analyzed by SDS-PAGE followed by Coomassie staining. Where indicated, dec-RVKR-cmk (20 µM) was added to the 1:1 molar ratio reaction to block furin activity.

The WT construct exhibited a high proteolytic activity against the Abz-Arg-Val-Lys-Arg-Gly-Leu-Ala-Tyr(NO_2_)-Asp-OH (Figure 3A) and Pyr-Arg-Thr-Lys-Arg-methyl-coumaryl-7-amide peptide substrates (data not shown). A specific inhibitor of furin (dec-RVKR-cmk) fully blocked the substrate cleavage by the WT enzyme. The correctly processed insertion mutants demonstrated low specific activity against the peptide substrates. A 1000-fold increase in the levels of the mutants in the cleavage reactions relative to that of the WT furin was required to detect their measurable activity.

To corroborate our observations, we tested the ability of the mutants to specifically cleave anthrax protective antigen-83 (PA83), a target that is highly sensitive to furin proteolysis [28], [29]. PA83 is known to be cleaved by furin at the Arg-Lys-Lys-Arg^196^ site [30], [31]. This cleavage converts PA83 into the PA63 functionally active species. Here, PA83 was subjected to proteolysis by increasing concentrations of the purified furins. PA83 was efficiently cleaved by the WT furin at an enzyme-substrate molar ratio as low as 1:10,000 (Figure 3B). A furin inhibitor dec-RVKR-cmk fully blocked the proteolysis of PA83 by furin. In contrast to the WT, the insertion mutants demonstrated very low activity against PA83. According to our tests, the mutants were 1,000–10,000-fold less potent against PA83 when compared to the WT enzyme. Based on these results we hypothesized that, after the prodomain excision the mutants remained self-inhibited by the N-terminal region of the mature enzyme which still occupied their active site.

### Interactions of furins with α1-PDX

**Figure 4:**
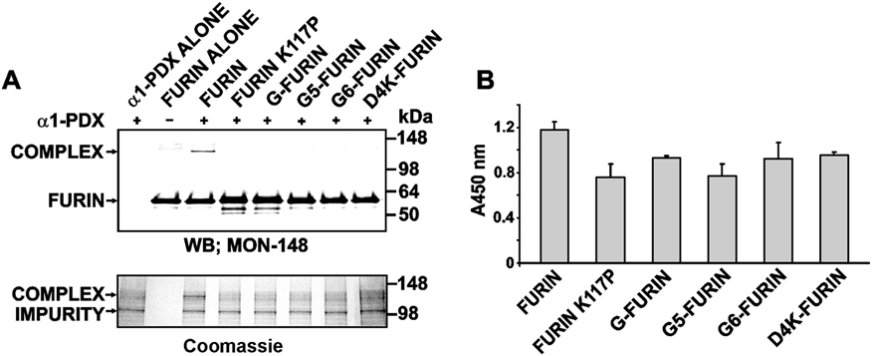
Binding of mutant furins with α1-PDX. (A) Stable complex formation between α1-PDX and furin. α1-PDX (5 µg) and furins (500 ng each) were co-incubated for 15 min. The reactions were stopped by 5 mM EDTA and 2% SDS, heated for 3 min at 95°C and then a 1/10 part of each reaction was analyzed by Western blotting with the MON-148 antibody against the catalytic domain of furin (upper panel) and a 9/10 part was used in SDS-PAGE followed by Coomassie staining (lower panel). Note the presence of the kinetically trapped, SDS-resistant α1-PDX complex with the WT furin. (B) ELISA of non-covalent complexes between α1-PDX and furins. The wells of a 96-well plate were coated with purified furins. FLAG-tagged αl-PDX was allowed to bind immobilized furins. The levels of the resulting complex were determined using a FLAG antibody followed by peroxidase-conjugated donkey anti-mouse IgG and a TMB/E substrate.

Normally, α1-PDX forms a covalent, SDS- and heat-stable, complex with functionally active furin [13]. To determine if the mutants were able to form a complex with α1-PDX, we co-incubated furins with α1-PDX to generate α1-PDX-furin complexes. The residual furin activity was inhibited using EDTA and SDS, and then by heating the samples at 95°C. The reactions were then analyzed both by SDS-PAGE followed by Coomassie staining and Western blotting with the MON-148 antibody against the catalytic domain of furin. Figure 4A shows that α1-PDX formed a stable complex with the WT furin but not with the mutants.

We next employed ELISA to analyze, in greater detail, the ability of furins to form a complex with α1-PDX (Figure 4B). The wells of a 96-well plate were coated with the purified furins. α1-PDX tagged with a FLAG tag was then added to the wells. The levels of the bound α1-PDX were then measured using the FLAG antibody. We determined that the ability of the mutant constructs to interact with α1-PDX was comparable with that of the WT furin. These data suggested that because the multiple S sub-sites were available in the mutants they were capable of a complex formation with the α1-PDX serpin but because the abnormally positioned N-terminal sequence shielded and incapacitated the S′ sub-sites, the inactive were unable to transform the initial association with the serpin into a kinetically trapped SDS-stable complex.

### Tryptic proteolysis of the furin constructs

**Figure 5:**
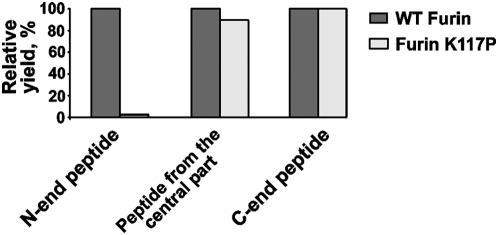
Mass spectrometry analysis of tryptic peptides. The K117P mutant and WT furin were subjected to a mild tryptic digest (trypsin-furin ratio of 1:100 and 1:1000 w/w, 5 min). The digests were analyzed by LC-MS/MS. The relative yield of the representative N-terminal (D^108^VYQEPTDPKFPQQWYLSGVTQR^130^) and the C-terminal (R^498^GDLAIHLVSPMGTR^512^) peptides and the peptide from the central part of the furin sequence (M^226^LDGEVTDAVEAR^238^) was determined using the differential peptide spectral count analysis. The yield of the WT peptides was taken as 100%. The data of the spectral count analysis of the selected peptides are summarized in Table S1.

To demonstrate that the N-terminal sequence is buried in the furin's catalytic cleft of the K117P mutant and exposed in WT furin, we used mild tryptic digestion. We suggested that the N-terminal peptides will be efficiently released by trypsin in WT furin but not in the K117P mutant. In turn, the release of the C-terminal peptides will be equally efficient in the WT and K117P constructs. The tryptic digest of the furin constructs followed by LC-MS/MS and the differential spectral count analysis of the peptides confirmed our suggestion. Thus, the release of the N-terminal D^108^VYQEPTDPKFPQQWYLSGVTQR^130^ peptide was 10–20 times more efficient in WT furin compared to that in the K117P mutant. To the contrary, the efficiency of the release of the C-terminal peptides including G^498^DLAIHLVSPMGTR^512^, the peptide from the central part of the furin sequence (M^226^LDGEVTDAVEAR^238^) and many addition tryptic peptides were highly similar in the WT and K117P furins (Figure 5). The data summarized in Table S1 confirm our conclusion that the N-terminal sequence of the emerging furin enzyme continues to occupy the catalytic cleft. As a result, this N-end sequence is protected from tryptic proteolysis, especially when compared to WT furin in which this N-terminal region is exposed and, therefore, can be easily accessed by trypsin.

### The residual levels of the inhibitory prodomain are associated with mutant furins

**Figure 6:**
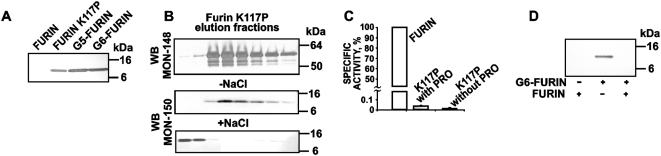
The presence of the residual levels of the intact prodomain in the mutant furin samples. (A) Western blotting of the purified furins. The purified furin samples (2 µg each) were analyzed by Western blotting with the MON-150 antibody against the furin prodomain. (B) The prodomain is separated from the activated K117P furin in the course of performing metal-chelating chromatography in the presence of 1 M NaCl. The K117P furin sample was purified by metal-chelating chromatography. The fractions eluted from the column using 500 mM imidazole were analyzed by Western blotting with the MON-148 antibody against the catalytic domain (*upper panel*) and with the MON-150 antibody against the prodomain of furin (*two bottom panels*). The 500 mM imidazole elution buffer did (+NaCl) or did not (-NaCl) contain 1 M NaCl. The presence of 1 mM NaCl had no effect on the elution profile of the K117P mature furin (*upper panel*). (C) Specific activity of the purified WT and K117P furins against Abz-Arg-Val-Lys-Arg-Gly-Leu-Ala-Tyr(NO_2_)-Asp-OH. The K117 mutant samples were purified either with or without 1 M NaCl and, therefore, the samples we tested either did or did not contain the residual levels of the prodomain (PRO). (D) The WT furin cleaves the prodomain observed in the G6-furin sample. The G6-furin sample (4 µg) which was purified in the absence of 1 M NaCl and therefore contained the residual amounts of the full-length prodomain, was co-incubated for 1 h at 37°C with purified furin (0.5 µg). The reactions were analyzed by Western blotting with the MON-150 antibody against the furin prodomain.

To exclude the possibility that the low proteolytic activity was due to the presence of the residual levels of the inhibitory prodomain in the furin samples, we evaluated the WT and K117P, G5 and G6 purified samples by Western blotting with the MON-150 antibody against the prodomain (Figure 6A). The WT samples did not show the presence of the prodomain. The purified mutants contained the residual levels of the 10 kDa prodomain. The molecular mass of the prodomain correlated with its full-length 27–107 sequence indicating that while the primary Arg-Thr-Lys-Arg^107^↓Asp^108^ cleavage site was autocatalytically processed the secondary cleavage site (Arg-Gly-Val-Thr-Lys-Arg^75^↓Ser^76^) remained intact in the mutants.

An additional analysis of the fractions eluted from the metal-chelating column in the course of the purification of the K117P mutant demonstrated that the prodomain was co-purified with the mature mutant enzyme (Figure 6B). The prodomain contamination, however, was successfully removed from the samples when the metal-chelating chromatography was performed in the presence of 1 M NaCl. We also demonstrated that the prodomain contamination did not affect the cleavage activity of the K117P mutant. Indeed, the activity of the K117P mutant samples which did and did not contain the prodomain remained similarly low (Figure 6C).

To verify that the mutant furins exhibited the full-length 27–107 prodomain, we co-incubated the G6-furin sample with the purified WT furin. The reactions were analyzed by Western blotting with the MON-150 antibody against the prodomain. As expected, the WT furin readily cleaved the secondary cleavage site (Arg-Gly-Val-Thr-Lys-Arg^75^↓Ser^76^) in the prodomain of the G6 sample and inactivated its immunoreactivity (Figure 6D).

### Prodomain cleavage *in trans*

**Figure 7:**
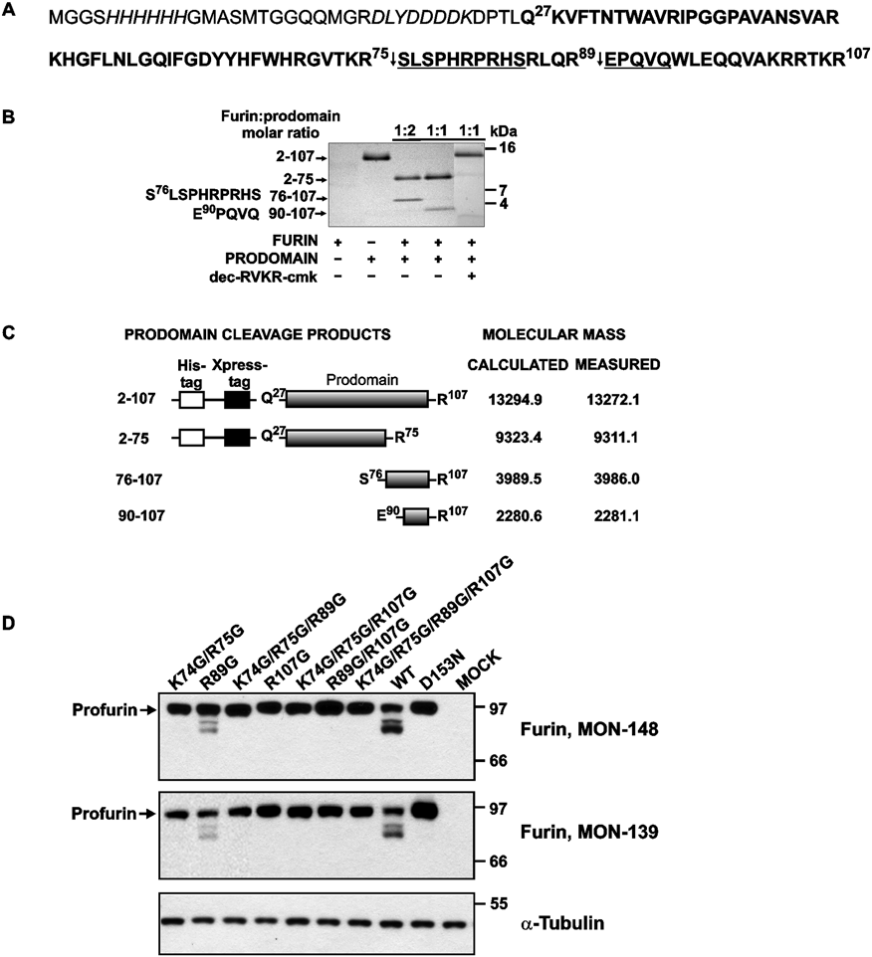
Furin proteolysis *in trans* of its inhibitory prodomain. (A) The recombinant furin prodomain was cloned into the pTrcHis A vector and expressed in *E. coli*. The sequence of the furin prodomain Gln27-Arg107 is in bold. The cleavage sites are indicated by the arrows. The N-terminal peptide sequence of the prodomain starts from Gly2 because of the cleavage of the initiating Met1 in *E. coli*. The sequence of the cleavage products determined by N-terminal sequencing is underlined. The sequence of the Xpress tag and His_6_ tag is italicized. The numbering used is for intact translated human furin. (B) The recombinant furin prodomain (1.8 µg) was incubated at 37°C for 1 h with furin at a molar ratio of 1:1 or 2:1. The digest samples were analyzed by Tricine gel electrophoresis followed by Coomassie staining. Where indicated, dec-RVKR-cmk (50 µM) was added. The sequence numbering of prodomain and cleavage products identified by MS analysis is shown on the left side. (C) The molecular mass of the intact recombinant furin prodomain and the cleavage products was determined by MALDI-TOF MS. The calculated and measured molecular mass of the peptides is shown in Da. (D) A Western blotting analysis of colon carcinoma LoVo cells transiently transfected with the WT furin (WT), the catalytically inert D153N mutant (D153N) and the furin mutants in which the primary (R107G), the secondary (K74G/R75G) and the tertiary (R89G) cleavage sites were inactivated in the prodomain sequence. The R89G/R107G; K74G/R75G/R89G and K74G/R75G/R107G, and K74G/R75G/R89G/R107G are the mutants in which two and three cleavage sites, respectively, were inactivated by mutations. Mock cells are LoVo cells transfected with the original plasmid. The antibodies to the catalytic domain and to the cytoplasmic tail of furin (MON-148 and MON-139, respectively) were used for Western blotting. To show equal loading, the samples were also analyzed by Western blotting with an α-tubulin antibody (bottom panel).

To gain additional insight into the proteolytic pathway that prevents re-inhibition of furin by the residual amount of its inhibitory prodomain (Figure 7A), we re-examined proteolysis of the prodomain sequence by furin. For this purpose, we subjected the purified individual prodomain sequence to furin proteolysis *in trans* (Figure 7B). Because the prodomain construct was expressed in frame with a His_6_ tag sequence and a short Xpress tag, the sequence of the actual prodomain commenced from Gln27 (preprofurin numbering). In contrast to the expected single cleavage of the 13 kDa tagged prodomain at the Arg-Gly-Val-Thr-Lys-Arg^75^↓Ser^76^ that should result in two peptide products [18], [19], [21], furin proteolysis of the prodomain followed by SDS-PAGE demonstrated the existence of two cleavage sites and the presence of three distinct products with apparent molecular weights of ∼10 kDa, ∼5 kDa and ∼3 kDa in the cleavage reactions. Dec-RVKR-cmk reversed the effect of furin and rescued the prodomain from furin proteolysis. N-terminal sequencing determined that the 5 kDa fragment commenced from Ser76 and therefore represented the 76–107 region of the prodomain. The determined N-terminal sequence (EPQVQ) suggested that the 3 kDa fragment represented the 90–107 C-terminal sequence of the prodomain (Figure 7C).

### Proteolytic processing of the prodomain in LoVo cells confirms a three-step mechanism

To confirm the importance of the cleavage at the tertiary cleavage site (Arg-Leu-Gln-Arg^89^↓Glu^90^) in the autocatalytic processing of profurin, we prepared the full-length WT furin and also the constructs in which the primary (R107G), the secondary (K74G/R75G) and the tertiary (R89G) cleavage sites were each inactivated by mutations. We also constructed the double (R89G/R107G, K74G/R75G/R89G and K74G/R75G/R107G) and the triple (K74G/R75G/R89G/R107G) furin mutants in which two and three cleavage sites, respectively, were inactivated by mutations. In addition, we prepared the full-length inert D153N furin. The constructs were transiently transfected in colon carcinoma LoVo cells. We specifically selected LoVo cells for these studies because of the two frameshift mutations in the furin gene of these cells do not allow them to produce furin [32]. Transiently transfected cells were lysed and the lysates were analyzed by Western blotting using the antibodies against the catalytic domain and the cytoplasmic tail of furin (MON-148 and MON-139, respectively). The WT furin was efficiently activated in LoVo cells and generated the intermediate and the mature enzyme (Figure 7D). The efficiency of self-activation of the R89G mutant in which the tertiary cleavage site (Arg-Leu-Gln-Arg^89^↓Glu^90^) was inactivated, was significantly reduced compared to that of WT furin. The inert mutant (D153N), the primary (R107G) and the secondary (K74G/R75G) cleavage site mutants as well as the double and triple mutants (K74G/R75G/R89G, K74G/R75G/R107G, R89G/R107G, K74G/R75G/R89G/R107G) were incapable of self-activation and, as a result, only the profurin form was detected in the corresponding cell lysates (Figure 7D). Based on these results, we conclude that the cleavage at the tertiary cleavage site (Arg-Leu-Gln-Arg^89^↓Glu^90^) and a three-step processing is required for the efficient autocatalytic processing and inactivation of the furin inhibitory prodomain *in vivo*. We suggest that this extensive, three-step processing helps the emerging furin to escape re-inhibition by the residual levels of the non-covalently associated prodomain.

## Discussion

To prevent unwanted protein degradation and to enable spatial and temporal regulation of proteolytic activity, proteolytic enzymes are synthesized as latent precursors [9], [33]. Proteolytic removal of the activation segment (which is also called the inhibitory prodomain) is required to generate a mature, functionally potent, enzyme. The prodomain size ranges from short peptide sequences to independently folded domains over 100 residues. In the multiple proteinases in which the preformed active site exists in the latent precursor, the prodomain sterically blocks the active site, and thereby prevents binding of substrates. In addition to their inhibitory role, the prodomain regions are frequently important for the folding, stability, and intracellular sorting of the latent precursor. Conversion to an active enzyme is stimulated by a change in pH that results in conversion by an autocatalytic mechanism. The requirement of the autocatalytic mechanism is well established for furin, the ubiquitous proprotein convertase that plays a highly significant role in the proteolytic processing of numerous functionally important proteins and peptides. Similar mechanisms are involved in the self-activation of PCs distinct from furin. The complexity of subcellular trafficking and the role of the associated factors (eg. heparan sulfate proteoglycan) in the regulation of PC activation, however, are not completely understood [34].

Generally, activation of furin requires several steps subsequent to the initial cleavage of its inhibitory N-terminal propeptide. As a result of these cleavages, the propeptide inhibitory activity is inactivated and the propeptide cleavage fragments are released from the active site of the emerging active furin enzyme [2], [14], [15], [16], [17], [18], [19], [21]. Earlier studies of the furin activation pathway have primarily been focused on processing the N-terminal prodomain thus leaving the structural re-arrangements of the N-terminal region of the emerging enzyme incompletely understood [7], [19], [21], [35]. Because the atomic resolution structures of the catalytic domain of furin and the prodomain of furin-like PC1 are known [22], [25], [36], we modeled the interactions of the prodomain and the catalytic domain which are characteristic for profurin and the transitions of the structure that follow the autocatalytic processing at the primary Arg-Thr-Lys-Arg^107^↓Asp^108^ site. It appears from our *in silico* modeling that the relative position of the N-terminal region of the catalytic domain is distinct in profurin and in mature furin. Following the cleavage at the primary site Arg-Thr-Lys-Arg^107^↓Asp^108^, the N-terminal region moves away from its original position in profurin to reach its final position in the mature furin molecule.

**Figure 8:**
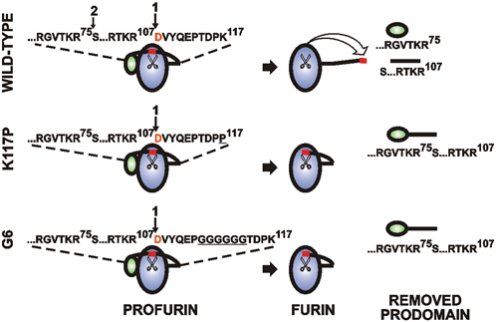
Self-activation of proproteinases requires the relocation of the N-terminus. Schematic representation of the autolytic activation mechanism of profurin. The 1 and 2 arrows above the protein sequence indicate the primary and secondary cleavage sites. The N-terminal Asp108 is shown in red. The mutant positions are underlined. The mutants do not perform the secondary cleavage and as a result the full-length prodomain sequence is liberated. This is in contrast to the WT processing which involves both the primary and the secondary cleavages of the prodomain and, as a result, two short fragments are generated. The repositioning of N-terminus is inactivated in the K117P and the G6 mutants.

According to our modeling, the electrostatic interactions of Asp115 with the calcium ion are especially important for the re-positioning of the N-terminal 108–113 region. The electrostatic interactions involving Asp115 and the calcium ion perform like a “loaded spring” and this spring is released after the pro-region cleavage to facilitate re-positioning of the N-terminal region from its original position in the furin zymogen to its final position in the mature, processed, furin. Our modeling also suggested that the peptide insertions provide an opportunity for the N-terminal part of the emerging furin to remain largely immobile after the autocatalytic cleavage at Arg-Thr-Lys-Arg^107^↓Asp^108^ and to continue to occupy the active site cavity in the mutants. As a result, the release mechanism remained inactivated and, we suspect, the mutants remained self-inhibited by the mature furin N-end region which continued to occupy the S′ positions of the catalytic cleft (Figure 8A).

To investigate these suspicions and to elucidate the auto-inhibitory function of the N-terminal region in greater detail, we studied the profurin mutants with the K117P mutation of the hinge motion Lys117 and the one, five and six residue insertions between positions Pro113 and Thr114. We determined that similar to the WT furin, these mutant furins were autocatalytically processed at the primary Arg-Thr-Lys-Arg^107^↓Asp^108^ site yielding the mature enzyme commencing from the N-terminal Asp108. In contrast to the WT construct the purified mutants, however, have low specific activity against the fluorescent peptide substrate and anthrax PA83, one of the most sensitive furin substrates. Our additional in-solution and ELISA binding assays confirmed that although the mutants, in contrast to normal furin, were incapable of forming an SDS- and heat stable complex with the α1-PDX serpin, they did bind the serpin non-covalently.

Tryptic digest of WT and mutant furins followed by LC-MS/MS and the differential peptide spectral count analysis of the tryptic fragments confirmed our suggestion that in the K117P mutant the N-terminal region of the emerging furin enzyme continued to be buried in the furin's catalytic cleft. As a result, this N-terminal sequence of the mutant was inaccessible to trypsin, especially when compared to WT furin.

Our *in vitro* cleavage and cell-based studies also identified an autocatalytic cleavage of the prodomain at Arg-Leu-Gln-Arg^89^↓Glu^90^ that is in addition to the primary (Arg-Thr-Lys-Arg^107^↓Asp^108^) and secondary (Arg-Gly-Val-Thr-Lys-Arg^75^↓Ser^76^) sites. Based on our results, we now believe that a three-step processing of the auto-inhibitory prodomain is required for its inactivation and for preventing re-inhibition of the emerging furin enzyme by the residual amounts of the non-covalently associated inhibitory prodomain. These results add another level of complexity to the molecular mechanics that are involved in the stepwise autocatalytic processing of the prodomain and imply a role for the auxiliary proteins and associated factors in these mechanisms [34].

Collectively, our results describe the events leading to the activation of furin and shed additional light on the restrictive role which is played by the enzyme's N-terminal region in the autocatalytic activation mechanisms of this and potentially other self-activated proteinases.

## Materials and Methods

### Materials

Reagents were purchased from Sigma-Aldrich unless indicated otherwise. The fluorescent substrate pyroglutamic acid-Arg-Thr-Lys-Arg-methyl-coumaryl-7-amide was obtained from Peptides International. The internally quenched fluorogenic peptide substrate Abz-Arg-Val-Lys-Arg-Gly-Leu-Ala-Tyr(NO2)-Asp-OH and the furin inhibitor dec-RVKR-cmk were from Bachem. Anthrax protective antigen-83 (PA83) was purchased from List Biological Laboratories. FLAG-tagged furin inhibitor α1-anti-trypsin variant Portland (α1-PDX) was from Affinity BioReagents. Restriction enzymes were from New England Biolabs. Sf9 insect cell line (an ovarian cell line from fall armyworm *Spodoptera frugiperda*), Cellfectin Reagent and a pcDNA 3.1 Directional TOPO Expression Kit were obtained from Invitrogen. The baculovirus transfer vector pAcGP67-A and the BaculoGold Linearized DNA were from BD Biosciences-Pharmingen. The Fugene HD transfection reagent was from Roche Diagnostics. Sf9 insect cells were routinely grown in Sf-900 II SFM medium supplemented with penicillin-streptomycin (100 units/ml and 100 µg/ml, respectively) and Fungizone/amphotericin B (0.25 µg/ml; Omega). The murine MON-148, MON-150 and MON-139 monoclonal antibodies against the catalytic domain, the prodomain and the cytoplasmic tail of furin, respectively, were from Axxora. The rabbit α-tubulin antibody was from Cell Signaling Technology. The peroxidase-conjugated donkey anti-mouse and anti-rabbit IgG were from Jackson ImmunoResearch Laboratories. The TMB/M and TMB/E substrates were purchased from Chemicon.

### Cloning of human furin and plasmid construction for the baculovirus expression system

Furin was amplified by PCR from a Marathon-Ready cDNA library (Clontech) with the 5′-ATGGAGCTGAGGCCCTGGTTGC-3′ and 5′-CTCATCAGAGGGCGCTCTGGTC-3′ oligonucleotides as direct and reverse primers, respectively. The resulting 2.4 kb cDNA fragment was cloned into the *HincII* site of the pUC18 plasmid and then recloned into the pcDNA3-zeo plasmid pre-digested with *HindIII* and *EcoRI*. The furin cDNA was then used as a template for preparing the furin constructs. Soluble furin was truncated at the position Ala574 (numbering starts from the initiation Met-1 of pre-profurin) and then C-terminally tagged with a Gly_3_-Ser-Gly_3_ linker followed by a His_6_ tag. The 5′-TGATAGCCCGGGCAGGGCCAGAAGGTCTTCACCAACACGTGGGCTGTGCGCATC-3′ and the 5′-AGGTATACACGCGGCCGCTTAATGGTGATGATGATGGTG *ACCTCCCCCAGAACCTCCCCC*GGCGGTGCCATAGAGTACGAG-3′ oligonucleotides were used as direct and reverse primers, respectively, in the PCR reactions (the His_6_ tag sequence is underlined and the linker sequence is italicized).

In the primers shown below the mutant positions are underlined. The K117P mutant (furin K117P) was generated by PCR mutagenesis using the 5′-TACCAGGAGCCCACAGACCCCCCGTTTCCTCAGCAGTGGTACCTG-3′ and 5′-CAGGTACCACTGCTGAGGAAACGGGGGGTCTGTGGGCTCCTGGTA-3′ forward and reverse primers, respectively. The D153N catalytically inert furin (furin D153N) with the substitution of the essential active site Asp153 was generated using the 5′-GGCATTGTGGTCTCCATTCTGAACGATGGCATCGAGAAGAACCAC-3′ and 5′-GTGGTTCTTCTCGATGCCATCGTTCAGAATGGAGACCACAATGCC-3′ forward and reverse primers, respectively.

To construct the insertion mutants, the Gly (G), Gly-Gly-Gly-Gly-Gly (G5), Gly-Gly-Gly-Gly-Gly-Gly (G6), His-His-His-His-His-His (H6) and Asp-Asp-Asp-Asp-Lys (D4K) sequences were each inserted by PCR mutagenesis between the positions Pro113 and Thr114 of the furin sequence. The G mutant (G-furin) was constructed using the 5′-GGGACGTGTACCAGGAGCCCGGCACAGACCCCAAGTTTCCTCA-3′ and 5′-TGAGGAAACTTGGGGTCTGTGCCGGGCTCCTGGTACACGTCCC-3′ oligonucleotides as the forward and reverse primers, respectively. The 5′-GGGACGTGTACCAGGAGCCCGGCGGTGGCGGTGGCACAGACCCCAAGTTTCCTCA-3′ (forward) and the 5′-TGAGGAAACTTGGGGTCTGTGCCACCGCCACCGCCGGGCTCCTGGTACACGTCCC-3′ (reverse) primers were used to obtain the G5 mutant (G5-furin). The 5′-GGGACGTGTACCAGGAGCCCGGTGGCGGTGGCGGTGGCACAGACCCCAAGTTTCCTCA-3′ (direct) and the 5′-TGAGGAAACTTGGGGTCTGTGCCACCGCCACCGCCACCGGGCTCCTGGTACACGTCCC-3′ (reverse) primers were used to prepare the G6 mutant (G6-furin). The His6 mutant (H6-furin) was constructed using the 5′-GGGACGTGTACCAGGAGCCCCATCACCATCATCATCACACAGACCCCAAGTTTCCTCA-3′ and 5′-TGAGGAAACTTGGGGTCTGTGTGATGATGATGGTGATGGGGCTCCTGGTACACGTCCC-3′ oligonucleotides as the forward and reverse primers, respectively. The 5′-GGGACGTGTACCAGGAGCCCGACGATGACGATAAGACAGACCCCAAGTTTCCTCA-3′ (direct) and the 5′-TGAGGAAACTTGGGGTCTGTCTTATCGTCATCGTCGGGCTCCTGGTACACGTCCC-3′ (reverse) primers were used to obtain the D4K mutant (D4K-furin). The resulting constructs were cloned into the *XmaI* and *NotI* sites of the pAcGP67-A vector. The authenticity of the constructs was confirmed by DNA sequencing.

### The wild-type and mutant furin constructs expressed in colon carcinoma LoVo cells

The full-length human furin cDNA was sub-cloned into the pcDNA 3.1/V5-His-TOPO using the 5′-CACCATGGAGCTGAGGCCCTGGTTGCTATGGGTGGTAGCAGCAACAGGAACCTT-3′ and the 5′-TCAGAGGGCGCTCTGGTCTTT-3′ oligonucleotides as forward and reverse primers, respectively. The furin mutants with the mutations in the primary (Arg-Thr-Lys-Arg^107^↓Asp^108^), secondary (Arg-Gly-Val-Thr-Lys-Arg^75^↓Ser^76^) and tertiary (Arg-Leu-Gln-Arg^89^↓Glu^90^) autocatalytic cleavage sites were generated by PCR mutagenesis using the wild-type furin template. The forward 5′-GTGGCAAAGCGACGGACTAAAGGGGACGTGTACCAGGAGCCCACA-3′ and reverse 5′-TGTGGGCTCCTGGTACACGTCCCCTTTAGTCCGTCGCTTTGCCAC-3′ primers were used to construct the primary cleavage site mutant (R107G). The forward 5′-TGGCATCGAGGAGTGACGGGGGGGTCCCTGTCGCCTCACCGC-3′ and the reverse 5′-GCGGTGAGGCGACAGGGACCCCCCCGTCACTCCTCGATGCCA-3′ primers were used to construct the secondary cleavage site mutant (K74G/R75G). The forward 5′-CGGCACAGCCGGCTGCAGGGGGAGCCTCAAGTACAGTGG-3′ and the reverse 5′-CCACTGTACTTGAGGCTCCCCCTGCAGCCGGCTGTGCCG-3′ primers were used to construct the tertiary cleavage site mutant (R89G). The double and triple mutants (K74G/R75G/R89G; K74G/R75G/R107G; R89G/R107G; K74G/R75G/R89G/R107G) in which two and three cleavage sites were inactivated by mutations were obtained by routine genetic engineering manipulations. The full-length D153N catalytically inert furin with the substitution of the essential active site Asp153 was generated using the 5′-GGCATTGTGGTCTCCATTCTGAACGATGGCATCGAGAAGAACCAC-3′ and 5′-GTGGTTCTTCTCGATGCCATCGTTCAGAATGGAGACCACAATGCC-3′ forward and reverse primers, respectively. The furin mutant constructs were cloned into the pcDNA 3.1/V5-His-TOPO and their sequences were confirmed by DNA sequencing.

Human colon carcinoma LoVo cells (ATCC) were grown in Dulbecco's modified Eagle's medium supplemented with 10% fetal bovine serum. Sub-confluent LoVo cells (5×10^5^) were transfected with 3 µl of Fugene HD reagent per 1 µg of DNA. Twenty four h after tranfection, cells were lysed in 50 mM Tris-HCl pH 8.0, supplemented with 1% SDS, 1 mM EDTA, 100 mM NaCl, 1 mM phenylmethyl sulfonylfluoride, and a protease inhibitor cocktail. The lysate samples (5 µg total protein each) were analyzed by Western blotting with the furin antibodies.

### Furin expression and purification

Sf9 cells were co-transfected with the recombinant pAcGP67-A vectors and the BaculoGold linearized DNA using Cellfectin. The amplification of the recombinant baculovirus and the preparation of a high-titer viral stock were performed in Sf9 cells according to the manufacturer's instructions (Invitrogen). Cells (2×10^6^ cell/ml) infected at a multiplicity of infection 1–2 were then cultured in suspension at 27°C in Sf-900 II SFM medium (1 L). After a 36–38 h incubation, the medium was centrifuged at 200×g and then at 15,000×g (30 min each; 4°C). The supernatant was concentrated 20-fold using an Amicon Ultra 30K-cutoff membrane (Millipore). 1 M Tris-HCl, pH 8.0, was added to the samples to obtain a final concentration of 20 mM. Furin was isolated from the medium samples using Ni^2+^-chelating chromatography on the HiTrap Chelating HP 1.6×2.5 cm size column (Amersham Biosciences) equilibrated with PBS. After eluting the impurities with 60 ml of a linear gradient of 0–25 mM imidazole, furin was eluted with 500 mM imidazole. Where indicated, 1 M NaCl was included in all of the buffers used for the purification of furin. The purified fractions were pooled, dialyzed against PBS and stored at -80°C. Purified furin samples were analyzed by SDS-PAGE, Western blotting and enzyme activity assays. The typical yield of purified furin was approximately 1 mg/liter of cell culture medium. Following SDS-PAGE and the transfer of the proteins onto a membrane support, the N-terminal peptide sequence of furin was determined by N-terminal microsequencing at ProSeq (Boxford, MA).

### The SDS- and heat-stable complexes of furin with α1-PDX

For the SDS-gel analysis followed by Coomassie staining, α1-PDX (5 µg, 10 µM) was co-incubated for 15 min at ambient temperature with furin (0.5 µg, 1 µM) in either PBS or the Sf-900 II SFM medium. For the Western blot analysis, the concentrations of α1-PDX and furin in the reactions were 10-fold less (0.5 µg and 50 ng, respectively). Furin activity was blocked by adding 5 mM EDTA and the 2× SDS-PAGE sample buffer (125 mM Tris-HCl, pH 6.8, 4% SDS, 0.005% Bromophenol Blue, 20 mM DTT and 20% glycerol) to the reactions. The reactions were heated for 3 min at 95°C and then analyzed by SDS-PAGE in a 4–20% acrylamide gradient gel followed by either Coomassie staining or Western blotting of the reaction aliquots with the MON-148 antibody.

### ELISA of the furin-α1-PDX complexes

To measure the efficiency of the α1-PDX binding with furin, the wells of a 96-well plate (Nunc) were coated for 16 h at 4°C with purified furin constructs (1 µg/ml in 100 µl PBS). BSA (1 µg/ml 100 µl PBS) was used as a control. The wells were then blocked with 3% BSA in PBS-0.05% Tween 20 for 2 h at the ambient temperature. α1-PDX (50 µg/ml in 100 µl PBS containing 3% BSA and 0.05% Tween 20) was added to the wells and the plates were incubated for 1 h at the ambient temperature. After washing the wells five times with PBS-0.05% Tween 20, the murine FLAG M2 antibody (0.5 µg/ml in 100 µl PBS containing 3% BSA and 0.05% Tween 20) was added to the wells and the plates were incubated 1 h at the ambient temperature. The unbound antibody was removed by washings with PBS-0.05% Tween 20. The peroxidase-conjugated donkey anti-mouse IgG (1:10,000 dilution) was added to the wells for 1 h. Following extensive washing with PBS-0.05% Tween 20 and then with water, a TMB/E substrate was added to the wells. The reaction was stopped by adding 0.1 ml 1 M H_2_SO_4_. The A_450_ value was measured using a plate reader. The samples were analyzed in triplicate.

### Purification of the furin prodomain

The prodomain was prepared using PCR amplification of the full-length furin cDNA. The 5′-CAGAAGGTCTTCACCAACACG-3′ and 5′-TCACCGTTTAGTCCGTCGCTTTGC-3′ oligonucleotides were used as direct and reverse primers, respectively, in the PCR reactions. The amplified fragment contained the prodomain sequence from Gln27 to the Arg-Thr-Lys-Arg^107^↓ primary cleavage site. The PCR fragment was subcloned into the pTrcHis A vector (Invitrogen). The resulting protein was expressed in frame with a His_6_ tag sequence, a linker region and an additional Xpress short tag**.** One Shot TOP 10 *E.coli* cells (Invitrogen) were transformed with the recombinant prodomain construct. The expression of the recombinant protein was induced with 1 mM isopropyl-1-thio-β-D-galactopyranoside. The collected cells were lysed, and the recombinant prodomain was purified from the cell lysates using Ni^2+^-chelating chromatography and reverse-phase HPLC. The purity of the recombinant prodomain construct was confirmed by SDS-PAGE and mass spectrometry.

### Cleavage of the furin prodomain

The furin prodomain (1.8 µg, 3.4 µM) was co-incubated with the purified furin samples (an enzyme-substrate ratio of 1:1 and 1:2) for 1 h at 37°C in 100 mM Hepes, pH 7.5, containing 1 mM CaCl_2_ and 1 mM β-mercaptoethanol. Where indicated, dec-RVKR-cmk (50 µM) was added to the reactions. The molecular mass of the intact prodomain and the digest products was determined by MALDI-TOF mass spectrometry on a Brucker Daltonics Autoflex II TOF/TOF mass spectrometer. To determine the N-terminal peptide sequence of the cleavage products and, consequently, the scissile bond, the reactions were separated by Tricine/SDS-PAGE in a 10–20% polyacrylamide gradient gel followed by the transfer of the digest fragments onto a membrane support. The Coomassie stained bands were excised and their N-terminal sequence was determined by N-terminal microsequencing at ProSeq.

### Tryptic proteolysis of furin and mass spectrometry

The purified WT and K117P furins (0.4 mg/ml each) were co-incubated for 5 min at 37°C in with trypsin (Promega) at a trypsin-furin ratio of 1:100 and 1:1000 (w/w) in PBS. Trypsin was then inactivated by 1 mM phenylmethyl sulfonylfluoride. The digest peptides were analyzed by LC-MS/MS using an Eksigent Nano 2D-LC system coupled with LTQ mass spectrometer (Thermo Scientific). The spectra were searched using Sorcerer SEQUEST (Sage-N Research) and filtered by PeptideProphet (Institute for Systems Biology; http://peptideprophet.sourceforge.net). Only the peptides with a probability score that exceeded 0.95 and a cross-correlation (Xcorr) value that exceeded 2.0 were further considered. The yield of the tryptic peptides was measured using the differential peptide spectral count analysis [37], [38].

### Enzymatic assays

Furin activity was measured in wells of a 96 well plate in 0.2 ml 100 mM Hepes, pH 7.5, containing 1 mM CaCl_2_, 1 mM β-mercaptoethanol and 0.005% Brij35. Abz-Arg-Val-Lys-Arg-Gly-Leu-Ala-Tyr(NO_2_)-Asp-OH (5 µM) was used as a substrate. The concentration of the wild-type (WT) and mutant furin in the reactions was 1 nM and 1 µM, respectively. The steady-state rate of substrate hydrolysis was monitored continuously (λ_ex_ = 320 nm and λ_em_ = 420 nm) at 37°C using a fluorescence spectrophotometer. When the Pyr-Arg-Thr-Lys-Arg-methyl-coumaryl-7-amide substrate was used, the steady-state rate was monitored at λ_ex_ = 360 nm and λ_em_ = 465 nm. The samples were measured in triplicate.

### Furin proteolysis of anthrax PA83

PA83 (1 µg, 1.2 µM) was co-incubated with furin at the indicated enzyme-substrate ratio for 1 h at 37°C in 100 mM Hepes, pH 7.5, containing 1 mM CaCl_2_ and 1 mM β-mercaptoethanol. The reactions were stopped by adding 2× SDS-PAGE sample buffer (125 mM Tris-HCl, pH 6.8, 4% SDS, 0.005% Bromophenol Blue, 20 mM DTT and 20% glycerol) to the samples. The digest reactions were analyzed in an 8–16% polyacrylamide gradient gel.

### Structure modeling

The structure of the human WT and mutant profurin was modeled using the available coordinates of the highly homologous (97% identity) catalytic domain and P-domain of murine 108–574 furin (Protein Data Bank code 1P8J) and the prodomain of murine 26–107 PC1 (37% identity; Protein Data Bank code 1KN6) as templates, and the Modeler software (http://ffas.ljcrf.edu). The human furin propeptide was docked into the crystal structure of the mouse furin catalytic domain using the DOT program (www.sdcs.edu/CCMS/DOT). To avoid steric clashes the several side chain positions were corrected manually using the Xfit program (www.sdcs.edu/CCMS/Packages/XTALVIEW/xtalview.html). The structure was then energy-minimized using the CHARMm22 force field molecular dynamics software of the Discovery Studio software package (Accelrys, http://www.accelrys.com/products/dstudio). The model was then validated using Molprobity (molprobity.biochem.duke.edu).

## Supporting Information

Table S1 (0.05 MB DOC)
