# Supplementary material for: Autocatalytic Activation of the Furin Zymogen Requires Removal of the Emerging Enzyme's N-Terminus from the Active Site
Source: PLoS One. 2009 Apr 7;4(4):e5031. doi: 10.1371/journal.pone.0005031 (PMC2662429; doi:10.1371/journal.pone.0005031)
Supplement: Table S1 — (0.05 MB DOC) [file pone.0005031.s001.doc]

**Table S1.** **Mass spectrometry analysis of the tryptic peptides of the WT and K117P furins.** Because of the K^117^/P^117^ mutation, the D^108^VYQEPTDPK^117^ and F^118^PQQWYLSGVTQR^130^ tryptic peptides of the K117P mutant cannot be generated and, therefore, these peptides were absent in the K117P samples.

| **Tryptic peptides** | **Trypsin-furin ratio (w/w)** | | | |
| --- | --- | --- | --- | --- |
|  | **1:1000** | | **1:100** | |
|  | **WT** | **K117P** | **WT** | **K117P** |
| **D^108^VYQEPTDPKFPQQWYLSGVTQRDLNVK^135^** | **=100** | **0** | **=100** | **0** |
| **D^108^VYQEPTDPKFPQQWYLSGVTQR^130^** | **=100** | **9** | **=100** | **0** |
| **D^108^VYQEPTDPK^117^** | **-** | **-** | **=100** | **0** |
| **F^118^PQQWYLSGVTQR^130^** | **=100** | **0** | **=100** | **0** |
| **A^136^AWAQGYTGHGIVVSILDDGIEK^158^** | **=100** | **100** | **=100** | **83** |
| **N^159^HPDLAGNYDPGASFDVNDQDPDPQPR^185^** | **=100** | **200** | **=100** | **200** |
| **Y^186^TQMNDNRHGTRCAGEVAAVANNGVCGVGVAYNAR^220^** | **=100** | **0** | **-** | **-** |
| **Y^186^TQMNDNR^193^** | **=100** | **0** | **=100** | **100** |
| **C^198^AGEVAAVANNGVCGVGVAYNAR^220^** | **=100** | **80** | **=100** | **111** |
| **I^221^GGVRMLDGEVTDAVEAR^238^** | **=100** | **94** | **-** | **-** |
| **M^226^ LDGEVTDAVEAR^238^** | **=100** | **100** | **=100** | **94** |
| **S^239^LGLNPNHIHIYSASWGPEDDGKTVDGPAR** | **=100** | **60** | **=100** | **89** |
| **S^239^LGLNPNHIHIYSASWGPEDDGK^261^** | **-** | **100** | **=100** | **250** |
| **L^269^AEEAFFRGVSQGR^282^** | **=100** | **127** | **=100** | **0** |
| **L^269^AEEAFFR^276^** | **=100** | **60** | **=100** | **64** |
| **G^283^GLGSIFVWASGNGGR^298^** | **=100** | **50** | **=100** | **200** |
| **Q^350^IVTTDLR^357^** | **-** | **-** | **=100** | **75** |
| **D^392^MQHLVVQTSKPAHLNANDWATNGVGRK^419^** | **-** | **-** | **=100** | **100** |
| **D^392^MQHLVVQTSKPAHLNANDWATNGVGR^418^** | **=100** | **200** | **=100** | **171** |
| **P^403^AHLNANDWATNGVGR^418^** | **-** | **-** | **=100** | **67** |
| **R^498^GDLAIHLVSPMGTR^512^** | **-** | **-** | **=100** | **100** |
